# Cold-Responsive Regions of Paradigm Cold-Shock and Non-Cold-Shock mRNAs Responsible for Cold Shock Translational Bias

**DOI:** 10.3390/ijms20030457

**Published:** 2019-01-22

**Authors:** Anna Maria Giuliodori, Attilio Fabbretti, Claudio Gualerzi

**Affiliations:** Laboratory of Genetics, University of Camerino, 62032 Camerino, Italy; attilio.fabbretti@unicam.it

**Keywords:** protein synthesis, translation initiation, translational bias, cold acclimation, chimeric mRNAs

## Abstract

In *Escherichia coli*, the mRNA transcribed from the main cold-shock gene *cspA* is a thermosensor, which at low temperature adopts a conformation particularly suitable for translation in the cold. Unlike *cspA*, its paralogue *cspD* is expressed only at 37 °C, is toxic so cannot be hyper-expressed in *E. coli* and is poorly translated in vitro, especially at low temperature. In this work, chimeric mRNAs consisting of different segments of *cspA* and *cspD* were constructed to determine if parts of *cspA* could confer cold-responsive properties to cspD to improve its expression. The activities of these chimeric mRNAs in translation and in partial steps of translation initiation such as formation of 30S initiation complexes and 50S subunits docking to 30S complexes to yield 70S initiation complexes were analyzed. We show that the 5′ untranslated region (5′UTR) of *cspA* mRNA is sufficient to improve the translation of *cspD* mRNA at 37 °C whereas both the 5′UTR and the region immediately downstream the *cspA* mRNA initiation triplet are essential for translation at low temperature. Furthermore, the translational apparatus of cold-stressed cells contains trans-active elements targeting both 5′UTR and downstream regions of *cspA* mRNA, thereby improving translation of specific chimeric constructs at both 15 and 37 °C.

## 1. Introduction

The life style of *enterobacteriaceae* like *Escherichia coli* entails frequent shuffling between the homeostatic environment of the gastrointestinal tract of the animal host and highly erratic external conditions. Overall, bacteria have developed and perfected adaptive mechanisms allowing them to overcome the potentially harmful consequences of the stresses (e.g., osmotic, nutritional, acid, temperature etc.) to which they respond with drastic structural and physiological changes and by extensive reprogramming of their gene expression patterns [1,2,3], which may also involve sRNAs [4]. Furthermore, the alterations occurring upon exposure to different types of stress may also impact human health, since stress-induced responses can play important roles in determining bacterial susceptibility to antibiotics [3] and expression of pathogenicity genes [5,6]. For instance, the expression of virulence genes can be under strict control of even subtle temperature variations such as the case of *Shigella* in which lowering the temperature by just a few degrees (i.e., below ≅ 32 °C) blocks the expression of the *virF* gene, which in turn controls the entire pathogenicity cascade. In this particular case, *virF* repression/activation occurs at the transcriptional level and is due to a slight change of the extent of DNA curvature near the promoter region and to a movement of the bending center which alternatively allows nucleoid protein H-NS or FIS to bind and to block or stimulate transcription, respectively [5,6].

Cold stress is among the most dramatic stresses that can affect *enterobacteriaceae.* Upon a temperature downshift from 37 to 20 °C or less, a large number of modifications occur in the *E. coli* cells which undergo a cold adaptation phase during which gene expression is reprogrammed as a result of both transcriptional and post-transcriptional regulatory events, whereby only a small set of cold-shock proteins is synthesized whereas bulk protein synthesis stops [7,8,9,10,11]. In this connection, an important role is played by a modification of the translational apparatus, which allows selective translation of cold-shock mRNAs and inhibition of non-cold-shock mRNAs translation during cold acclimation [12,13,14,15]. This phenomenon, designated “cold-shock translational bias” is due in part to trans-acting factors such as initiation factors IF3 and IF1, whose levels increase with respect to ribosomes [16,17] whose synthesis slows down [18] and in part to cis-acting elements present in the cold-shock transcripts [15,19].

Indeed, it has been shown that the mRNA encoding CspA, a single stranded nucleic acid binding protein, which is present in extraordinary large amounts in cells during early exponential growth [20,21] and also after cold stress [7,20,22,23], is endowed with a structure that functions as a temperature-dependent molecular switch. This property is due to the 5′UTR of *cspA* mRNA which at low temperature maintains a folding likely identical to that of transcriptional intermediates. In fact, *cspA* mRNA fragments <190 nts have an identical structure at 37, 20 and 10 °C. However, if the mRNA size exceeds 190 nts, the folding pathway diverges at 37 °C and at low temperature; at 37 °C, the 5′UTR associates with the coding region, whereas it remains folded independently of the rest of the molecule at low temperature. This structural variance is reflected in a difference at the level of the translation initiation region (TIR), which becomes more accessible to the ribosomes in the cold conformation [19]. Therefore, the structural change occurring upon lowering the temperature is responsible for the higher translational efficiency observed under cold stress conditions [9,11,12,15]. Remarkably, *cspA* mRNA is also translated at high level at 37 °C in its 37 °C conformation because cells accumulate large amounts of this protein during growth resumption and during the early stages of growth [20,21].

In *E. coli*, in addition to *cspA* there are nine other *csp* genes scattered within the chromosome, only five of which (*cspA, cspB, cspE, cspG* and *cspI*) are induced during cold-shock [24]. *CspD* is a monocistronic “non-cold-shock” gene that does not respond to temperature downshifts and is expressed only during the stationary-phase of cell growth or upon glucose starvation [25]. CspD inhibits DNA replication at both the initiation and the elongation stages and it is probably because of this activity that its overproduction causes cell death [25]. The exact mechanism which regulates CspD synthesis remains for the most part unknown. H-NS favors somewhat *cspD* expression after the entry of the cells into stationary growth phase [25] and the toxin-antitoxin complex MqsR/MqsA might control its transcription [26,27]. Also, the cyclic AMP receptor protein (CRP) positively regulates *cspD* transcription [28]. However, *cspD* expression is likely regulated also at the post-transcriptional level, like other *csp* mRNAs [1]. This assumption is supported by the fact that in vitro translation of *cspD* mRNA is modest at 37 °C and very poor at low temperature [15]. Furthermore, despite its superficial similarity to *cspA* mRNA in terms of size, sequence and overall design [12] our experimental evidence shows that the structure of *cspD* mRNA, unlike that of *cspA* mRNA, does not undergo a temperature-dependent structural transition and is characterized by a helix which sequesters the translation initiation triplet and is discriminated against by the translational apparatus of cold shocked cells [15,29]. 

In this article, we have constructed chimeric mRNAs by swapping different segments of the cold-shock *cspA* mRNA and the non-cold-shock *cspD* mRNA and tested their efficiency in directing the formation of intermediate complexes of the translation initiation pathway as well as their activity in promoting protein synthesis in vitro as a function of temperature and in the presence of a control and cold-shock translational apparatus. 

Our aim was to identify the *cspA* elements which could be used to transform an mRNA such as *cspD,* clearly refractory to translation, in particular at low temperature, into an mRNA with features similar to those of the cold-shock transcript. 

Here, we demonstrate that the 5′UTR and the region immediately downstream the initiation triplet of *cspA* mRNA are essential for translation at low temperature, whereas the remaining coding sequence does not affect translational efficiency. In addition, we show that the extracts prepared from cells subjected to cold-shock contain trans-acting factors which target these *cspA* mRNA regions and favor the translation of the chimeric constructs at both 37 and 15 °C, whereas the low translational efficiency of the mRNAs lacking these cis-elements can be explained by the formation of structurally unfit (non-canonical) 30S initiation complexes which at low temperature fail to dock to the 50S subunit to yield a productive 70S initiation complex. The understanding of these mechanisms may help to improve systems of in vitro protein synthesis.

## 2. Results

### 2.1. Construction of Chimeric mRNAs

**Figure 1:**
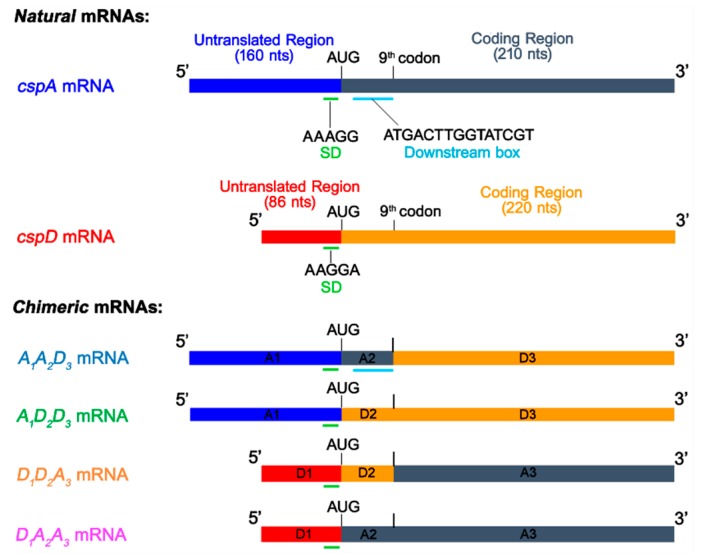
Schematic representation of the mRNAs prepared and used in this study. The two original mRNAs are shown at the top of the scheme: the 5′ untranslated regions (5′UTR) of *cspA* (blue) and *cspD* mRNA (red), which represent the RNA fragments A_1_ and D_1_, respectively, and the coding regions of *cspA* (dark gray) and *cspD* mRNA (orange) are colour coded. Also indicated are the positions of initiation triplets, SD sequence and downstream box (DB) as well as the position of the 9^th^ codon which represents the A_2_/A_3_ and D_2_/D_3_ borders between the RNA fragments derived from *cspA* and *cspD*, respectively. The lower part of the scheme shows the four mRNAs constructed by swapping the three RNA fragments (A_1_A_2_A_3_ and D_1_D_2_D_3_) of the mRNA to construct the chimeric mRNAs indicated on the left side of the figure. The construction of the chimeric mRNAs is described in Materials and Methods.

The mRNAs encoding CspA and CspD were selected as paradigm cold shock and non-cold shock mRNAs, respectively. As illustrated in the scheme (Figure 1), the sequences of these two mRNAs were divided in three segments, the first A_1_ (160 nts) and D_1_ (86 nts) correspond to their 5′ untranslated region (5′UTR); the second segments, A_2_ and D_2_ correspond to the first 9 codons which contain the so called “downstream box” (DB) which was claimed, at least in the case of *cspA* mRNA, to affect translational efficiency [30,31], especially at low temperature [32,33,34,35], while the last segments, A_3_ and D_3_, correspond to the remaining coding region of *cspA* and *cspD* mRNA, respectively. The DNA sequences were manipulated (Figure 1) so as to swap the various segments and obtain four chimeric mRNAs: A_1_A_2_D_3_, D_1_A_2_A_3_, A_1_D_2_D_3_, D_1_D_2_A_3_ whereas the original mRNAs (A_1_A_2_A_3_ and D_1_D_2_D_3_) were used as controls in the experiments in which chimeric mRNAs were tested in translational functions.

### 2.2. mRNAs-Dependent Translation in Cell Extracts of Control and Cold-Stressed Cells

**Figure 2:**
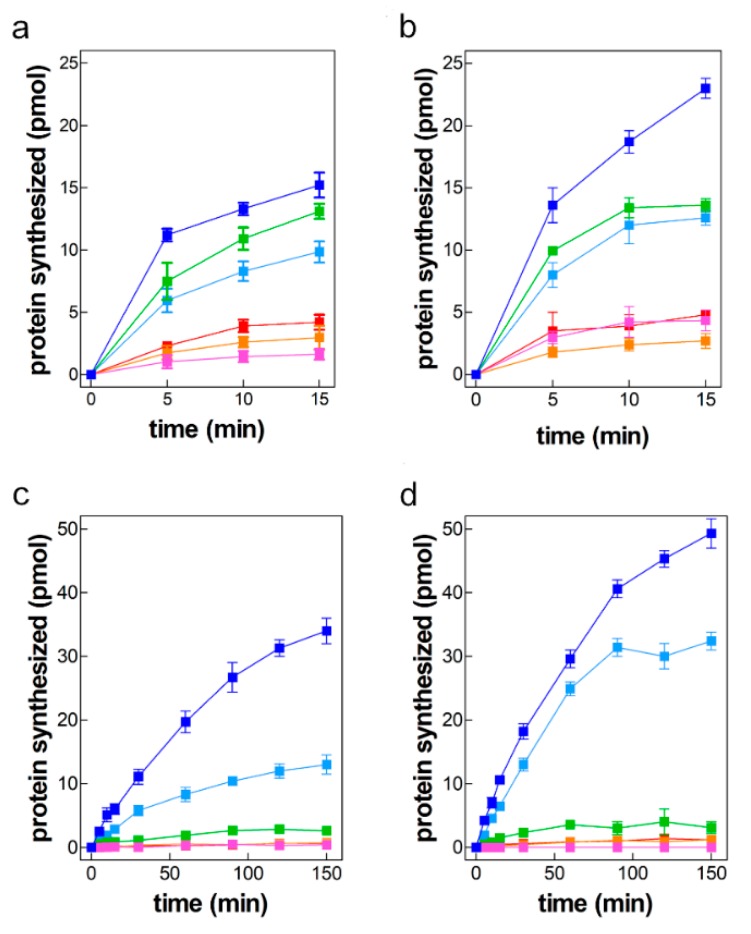
Translational activity of various mRNAs at 37 and 15 °C with cell extracts of control and cold-stressed cells. Time course of in vitro protein synthesis directed by mRNAs *cspA* (dark blue); *cspD* (red); A_1_A_2_D_3_ (light blue); A_1_D_2_D_3_ (green); D_1_A_2_A_3_ (magenta); D_1_D_2_A_3_ (orange) at 37 °C (**a**,**b**) and 15 °C (**c**,**d**) by cell extracts of control cells (**a**,**c**) and cells subjected to cold stress (**b**,**d**). Each data point is the mean of a duplicate and error bars indicate the standard deviation. The radioactivity incorporated in the absence of mRNA was taken as background and subtracted from each point. Further details are given in Materials and Methods.

The mRNAs described above were used to program extracts (S30) of control cells grown at 37 °C (Figure 2a,c) or of cold stressed (cs) (Figure 2b,d) cells. The translational activity at 37 °C of all mRNAs containing the 5′UTR of *cspA* mRNA (i.e., A_1_A_2_A_3_, A_1_A_2_D_3_ and A_1_D_2_D_3_) was found to be substantially higher than that of the mRNAs containing instead the 5′UTR of *cspD* mRNA (D_1_D_2_D_3_, D_1_D_2_A_3_, D_1_A_2_A_3_) (Figure 2a,b) with both types of extracts. Quite different results were obtained when translation was carried out at the cold shock temperature (i.e., 15 °C). In this case only the mRNAs containing both 5′UTR and the proximal region of the coding sequence proved to be translationally active whereas essentially no translation could be detected with the other mRNAs (Figure 2c,d). It is noteworthy that the translational activity of the A_1_A_2_D_3_ mRNA is almost as high as that of the *cspA* mRNA when translation was carried out with the extracts of cold shocked cells (Figure 2d) but substantially lower with the extracts of control cells (Figure 2c).

**Figure 3:**
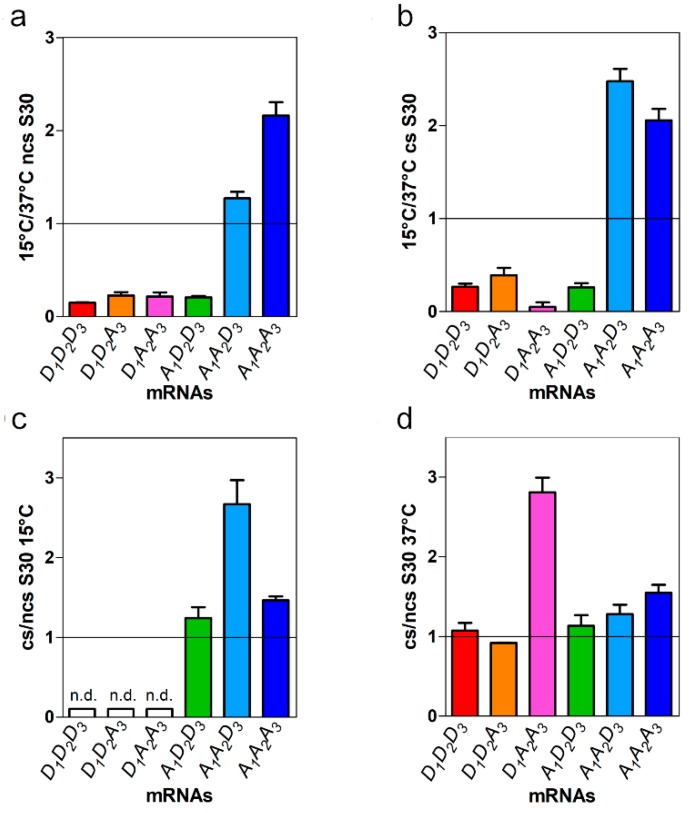
Relative levels of translation recorded for the various mRNAs as a function of temperature in the presence of the extracts of control and cold shocked cells. The histograms indicate the ratio of the translational level obtained with the mRNAs indicated in the abscissa at (**a**,**b**) 15 and 37 °C in the presence of extracts of control (**a**) and (**b**) cold-shocked cells. The ratios of the translational level obtained with the indicated mRNAs in cold shock and control cell extracts at (**c**) 15 °C and (**d**) 37 °C. Each histogram represents the average ratio calculated from a duplicate; error bars indicate the standard deviation. n.d.=not detected.

A comparison of the maximum level of product synthesized at 15 and 37 °C by S30 extracts of non-cold-shocked (ncs) cells programmed with the various mRNAs (Figure 3a) shows that, as a result of the capacity of *cspA* mRNA to assume a cold conformation particularly prone to translation in the cold, the amount of product synthesized with this template at 15 °C is higher than that synthesized at 37 °C (15 °C/37 °C ≅ 2.2). Likewise, the chimeric A_1_A_2_D_3_ mRNA maintains at least in part this property as the amount of product synthesized at 15 °C with this mRNA is slightly higher than that made at 37 °C (15 °C/37 °C ≅ 1.2). Unlike in these two cases, for all other mRNAs the 15 °C/37 °C product ratios were <<1 (Figure 3a). 

A similar comparison, performed for the products synthesized using the extracts of cold-shocked (cs) cells confirmed that only *cspA* and A_1_A_2_D_3_ mRNAs are translated more efficiently in the cold than at 37 °C, the 15 °C/37 °C ratio being ≥2 for these mRNAs and <<1 for all the other (Figure 3b). The presence of trans-acting factors which favor protein synthesis at low temperature in the translational apparatus of cold-stresses cell is indicated by the comparison of the level of translation obtained at 15 °C with a cell extract of cold-stressed cells and with an extract of control (i.e., non-stressed) cells. As seen in Figure 3c, the ratio of the translational level is >1 for all mRNAs containing the 5′UTR of *cspA* indicating that this portion of the mRNA represents the target of this/these trans-acting factor/s. On the other hand, this ratio cannot be reliably calculated for the D_1_D_2_D_3_, D_1_D_2_A_3_ and D_1_A_2_A_3_ mRNAs because at 15 °C the level of translation with these templates is barely detectable above the background (Figure 3c).

Taken together, these findings indicate that the 5′UTR and the proximal region of the *cspA* coding sequence are essential to allow translation at low temperature. The high translational efficiency at 15 °C of the mRNAs containing these elements (i.e., *cspA* mRNA and A_1_A_2_D_3_ mRNA) is likely due to an RNA conformation more favorable for translation at low temperature. Furthermore, the higher translational activity of the mRNAs containing the 5′UTR and the proximal region of the *cspA* coding sequence observed in the presence of the extracts of cold stressed cells indicates that these cells contain trans-acting factor(s) [15,16,17] targeting these RNA segments and favoring protein synthesis at low temperature. 

### 2.3. Kinetic Analyses of 30S Initiation Complex Formation

**Figure 4:**
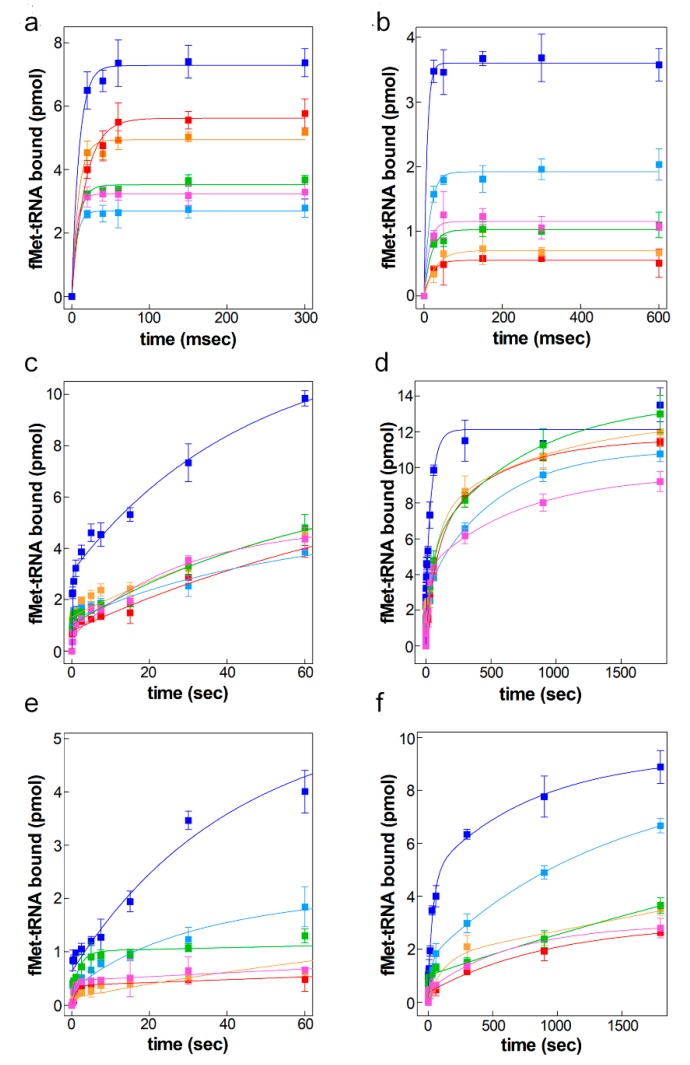
Kinetics of 30S initiation complex formation with various mRNAs. Kinetics of fMet-tRNA binding at (**a**) 37 °C and (**b**) 15 °C to (**a**,**b**) 30S subunits pre-incubated with different mRNAs and (**c–f**) upon offering simultaneously fMet-tRNA and the various mRNAs to the 30S subunits kinetics at (**c**,**d**) 37 °C and (**e**,**f**) 15 °C. Binding was followed by fast filtration on nitrocellulose using a quenched-flow apparatus and therefore measures the formation of a “locked” 30S IC. The 30S subunits contained a (1:1) stoichiometric equivalent of IF1, IF2–GTP and IF3 and the mRNAs used were: *cspA* (dark blue); *cspD* (red); A_1_A_2_D_3_ (light blue); A_1_D_2_D_3_ (green); D_1_A_2_A_3_ (magenta); D_1_D_2_A_3_ (orange). Each data point is the mean of quadruplicates; error bars represent the standard deviation. The radioactivity incorporated in the absence of mRNA was taken as background and was subtracted from each point. Further details are given in Materials and Methods.

To determine which translational step is influenced by the presence of the various RNA segments constituting the chimeric mRNAs, kinetics of fMet-tRNA binding to 30S ribosomal subunits programmed with the different types of mRNAs was studied by rapid filtration using a quench flow apparatus to monitor the most rapid phases (Figure 4). In this connection it must be recalled that rapid filtration allows detection of the formation of a *bona fide* “locked” 30S initiation complex (30S IC) in which mRNA and initiator tRNA are locked by proper codon-anticodon base pairing [36,37,38,39]. In the first set of experiments, binding of fMet-tRNA to 30S ribosomal subunits pre-incubated at 37 or 15 °C with the three initiation factors (IFs) and the various mRNAs was monitored at the same temperatures. At 37 °C fMet-tRNA bound very rapidly to all 30S-IFs-mRNA complexes (apparent rate constant >100/s for all mRNAs, but for *cspD* mRNA), albeit the binding levels differed in each case; the most efficient template proved to be *cspA* mRNA, followed by the *cspD* mRNA and by the D_1_D_2_A_3_ mRNA which yielded a ca. 25% lower binding whereas binding directed by the other three mRNAs was ≤50% lower (Figure 4a). *CspA* mRNA was the best template also at 15 °C, although the amount of fMet-tRNA bound in its presence was half that obtained at 37 °C (Figure 4b) while the complex formed with A_1_A_2_D_3_ mRNA was ca. 50% less than that formed under the same conditions with *cspA* mRNA. All the other mRNAs displayed a lower fMet-tRNA binding efficiency, the lowest activity being found with *cspD* mRNA and with D_1_D_2_A_3_ mRNA (Figure 4b). The only mRNA able to stimulate the binding of fMet-tRNA to the 30S subunit in comparable amounts at 15 and 37 °C was A_1_A_2_D_3_ mRNA (15 °C/37 °C ≅ 1) followed by *cspA* mRNA (15 °C/37 °C ≅ 0.5). 

The apparent rate constants (K_app_) for fMet-tRNA binding at 37 °C were found to be 2–3 times higher than those obtained at 15 °C for all mRNAs, the only exceptions being *cspA* and *cspD* mRNAs; in the first case the apparent binding rate at 15 °C was somewhat higher than that determined at 37 °C whereas with *cspD* mRNA the rates were equally low at both temperatures (Table 1). Overall, because the on-rates measured at the same temperature are rather similar for all mRNAs, but for the just mentioned case of *cspD* mRNA, it can be concluded that the different levels of fMet-tRNA binding observed with the various mRNAs at each temperature are most likely due to differences in the off-rates of the complexes and/or to differences in the amounts of 30S-IFs-mRNA complexes formed in each case during the pre-incubation step. Furthermore, it can be surmised from these results that neither the 5′UTR alone nor the whole coding sequence downstream the 5′UTR plays a role in determining the mRNA binding property at low temperature, but that only the 5′UTR of *cspA,* in combination with the proximal portion of the coding sequence, confers a good binding activity to the mRNAs at 15 °C.

In another set of experiments, the kinetics of “locked” 30S IC formation was monitored by mixing rapidly fMet-tRNA and the individual mRNAs with IFs-containing 30S subunits. Under these conditions the binding of the mRNA to the complex is rate limiting and fMet-tRNA binding is much slower than described above when the mRNA binding step was bypassed by a preliminary incubation of the mRNAs with the 30S subunits. As seen in Figure 4c,e, an at least 1 min incubation is required to obtain a level of fMet-tRNA bound comparable to that attained in less than 50 msec when 30S subunit and mRNA are pre-incubated. In this range of time at 37 °C, *cspA* mRNA is the most efficient template in terms of both level and rate of fMet-tRNA binding (Figure 4c). 

However, if the incubation at 37 °C is prolonged to 30 minutes all mRNAs yield comparable levels of fMet-tRNA binding (Figure 4d). The situation is completely different at 15 °C. In fact, while *cspA* mRNA remains the most efficient template as far as level and rate of fMet-tRNA binding at both short (Figure 4e) and long (Figure 4f) incubation times, all the other templates proved to be rather inefficient with the exception of A_1_A_2_D_3_ mRNA which yielded a higher level of fMet-tRNA binding than all the other mRNAs. However, it should be noted that the A_1_D_2_D_3_ mRNA is the one displaying the fastest rate of binding although it eventually yielded a lower level of binding. 

In addition, unlike the kinetics of fMet-tRNA binding to 30S-mRNA complexes (Figure 4a,b), which can be fitted by a single exponential equation (Table 1), these kinetics can be fitted by a two exponential or a three exponential equation, described by two or three apparent rate constants (K_1_, K_2_ and K_3_) yielding two or three maximum plateaux (Y_max1_ Y_max2_, and Y_max3_), respectively. This behavior likely reflects the complex nature of the reaction (Table 2). As seen from this Table, at 37 °C the reaction proceeds in two phases only for *cspA* mRNA, whereas a third slower phase, during which a large fraction of the complexes are formed, is present with all other mRNAs. However, it is likely that, due to its extreme slowness, this phase does not play any role during translation. 

With respect to the binding reaction at 15 °C, the low temperature impacts differently on the kinetics of 30S IC formation depending on the type of mRNA. The only complex formed in reasonably high amounts is that containing *cspA* mRNA which is formed with the intermediate kinetic rate because the fast phase of binding disappears at low temperature. The fast kinetic phase (~10/s) is still present in the formation of the complex containing A_1_A_2_D mRNA, although only a small fraction of the complex is formed at this rate and with A_1_D_2_D_3_ mRNA where the fast phase yields a higher amount of complex. These results confirm that the A_1_ and A_2_ elements of *cspA* confer upon the D_3_ region an improvement in terms of both kinetics (A_1_) and amount of 30S IC formed (A_1_ + A_2_), although none of the tested combinations perform as well as *cspA* mRNA. On the other hand, at 15 °C, *cspD* mRNA and the chimeric mRNAs containing the D_1_ and D_2_ elements form a complex either at a slower rate (lower K_app_) than the other mRNAs or in lower amounts (lower Y_max_).

Overall, the results of the binding kinetics are compatible with the results obtained in translation (Figure 2) insofar as they show that at 37 °C, all mRNAs promote, albeit at different levels, the formation of a sufficient amount of locked 30S complex and direct protein synthesis, whereas at 15 °C, only the mRNAs containing the 5′UTR and the proximal coding region of *cspA* (i.e., *cspA* mRNA and A_1_A_2_D_3_ mRNA) promote the formation of a substantial amount of 30S IC, which ultimately allows protein synthesis to occur. 

### 2.4. 50S Docking to the Various 30S Complexes 

During the translation initiation pathway, the formation of a 30S IC is followed by docking of the 50S ribosomal subunit to yield a 70S IC in which the fMet-tRNA eventually occupies the P-site where it can donate the formyl-Methionine to the incoming aminoacyl-tRNA carried to the A-site by elongation factor EF-Tu [40]. The docking represents a critical kinetic checkpoint under the control of IF3 and IF1 which ensures faithful translation initiation insofar as non-canonical 30S complexes are rejected at this stage [41]. 

**Figure 5:**
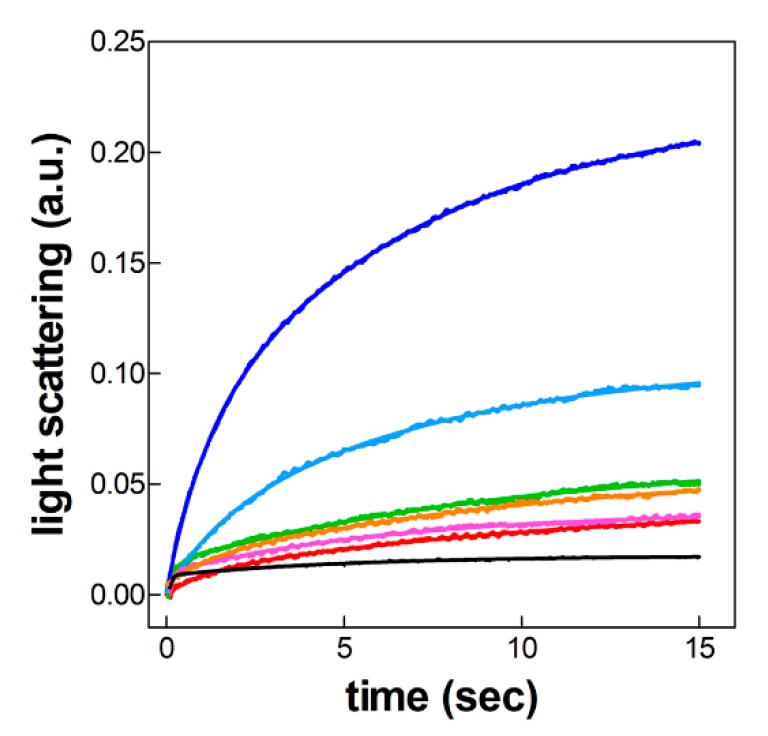
Kinetics of 50S subunits docking to 30S initiation complexes formed with different mRNAs. 30S initiation complexes, prepared by 30 min incubation at 15 °C using various mRNAs, were rapidly mixed in a stopped flow apparatus with a stoichiometric equivalent of 50S ribosome subunits and the real time change of the light scattering signal (expressed in arbitrary units in the ordinate) generated by subunits association was recorded. *cspA* (dark blue); *cspD* (red); A_1_A_2_D_3_ (light blue); A_1_D_2_D_3_ (green); D_1_A_2_A_3_ (magenta); D_1_D_2_A_3_ (orange). Each trace is the average of at least 15 different shots. The light scattering values detected at time = 0 were subtracted from each point of the traces. Further details are given in Materials and Methods.

Thus, in the following experiments, docking of the 50S subunit to the 30S-mRNA-fMet-tRNA complexes formed with the various mRNAs was analyzed by measuring the time-resolved variations of the light scattering signal generated by the formation of a 70S IC whose mass is far larger than that of the two isolated subunits. It can be seen that only the complexes containing *cspA* mRNA and, to some extent, A_1_A_2_D_3_ mRNA undergo successful docking with the 50S subunit at 15 °C, whereas all the others fail to form a sufficient amount of 70S IC (Figure 5). These data clearly indicate that only the mRNAs containing the 5′UTR and the proximal coding sequence of *cspA* mRNA give rise to 30S complexes endowed with a canonical structure, whereas the structures of the complexes containing all the other mRNAs are geometrically unfit for binding to the 50S subunit. In turn, this conclusion explains the reason why only *cspA* mRNA and, A_1_A_2_D_3_ mRNA are active in directing protein synthesis at low temperature (Figure 2c,d). 

## 3. Discussion

All mRNAs tested proved to be active at 37 °C, albeit to different extents, in promoting fMet-tRNA binding to the 30S ribosomal subunit and in directing protein synthesis in the presence of S30 extracts of either control or cold shocked cells. The translational efficiencies of the various mRNAs ranged in the order: *cspA* mRNA > A_1_D_2_D_3_ mRNA > A_1_A_2_D_3_ mRNA > *cspD* mRNA ≈ D_1_D_2_A_3_ mRNA ≈ D_1_A*_2_*A_3_ mRNA. 

To form a 30S initiation complex (30S IC), mRNA and fMet-tRNA are initially bound to the 30S subunit without fully interacting with each other; a subsequent first order isomerization of this 30S pre-IC, which entails a structural rearrangement of the GGAA (G1516–A1519) tetraloop of h45 that alters the h44/h45/h24a interface, allows complete codon-anticodon base pairing and produces a “locked” 30S IC [36,38]. 

Here, we have analyzed in two ways the formation of 30S IC in the presence of the various mRNAs. In one case, when the mRNA binding step was bypassed by pre-incubating the 30S subunit with the mRNAs, the rate of 30S IC formation was very fast and similar for all mRNAs (Table 1), indicating that the kinetics of the codon-anticodon interaction is only marginally affected by the nature of the mRNA, provided that the mRNA is pre-bound to the 30S subunit. However, the conditions of these experiments and the efficiencies displayed by the various mRNAs in promoting fMet-tRNA binding do not correspond to the natural conditions and the results obtained are different from the results of the translational tests at 37 °C.

Indeed, a completely different situation was observed when the “locked” 30S IC was formed by rapidly mixing IFs-bound 30S subunits with the mRNAs and fMet-tRNA. Under these conditions, the reaction kinetics are affected by the different structures of the mRNAs which make them more or less prone to bind rapidly and correctly to the ribosomal subunit and undergo the conformational transition which allows codon-anticodon base pairing yielding the 30S IC. In these cases, the equations fitting the experimental curves are multi-exponential, indicating the occurrence of two or three phases, depending on type of mRNA and temperature. Under these experimental conditions, the differences between the way *cspD*, D_1_D_2_A_3_, and D_1_A_2_A_3_, mRNAs on the one hand and *cspA* mRNA on the other promote 30S IC formation after short incubation times at 37 °C reflect more closely their differences in translational activity. These results suggest that the kinetics of locked 30S IC formation likely plays a major role in determining the translational efficiency of these mRNAs at 37 °C. 

Surprisingly, the A_1_A_2_D_3_ and A_1_D_2_D_3_ mRNAs perform very well in translation at 37 °C but are much less efficient in forming a locked 30S IC at the same temperature. This discrepancy indicates that the translation of these mRNAs may benefit from the melting of the secondary structures produced by the first ribosome moving on the mRNA, thereby favoring the formation of additional 30S IC complexes. 

Unlike the situation seen at 37 °C, at 15 °C only *cspA* and A_1_A_2_D_3_ mRNA were able to rapidly form a sufficient amount of 30S IC, whereas all the other mRNAs promoted formation of only a small amount of 30S complexes and only after a very long incubation time. These results match the translational activities since only *cspA* mRNA and A_1_A_2_D_3_ mRNA were able to direct protein synthesis at 15 °C, while none of the other mRNAs was translationally active at low temperature. 

Because *cspA* mRNA and A_1_A_2_D_3_ mRNA are the only templates which contain both 5′UTR and proximal coding region of *cspA* mRNA, it can be concluded that these two elements are necessary and sufficient to ensure 30S IC formation and translation at low temperature. Furthermore, the large increase of the translational activity of *cspA* mRNA and, even more remarkably, of A_1_A_2_D_3_ mRNA observed when the control S30 is replaced by the S30 derived from cold shocked cells indicates that these two regions of the *cspA* mRNA are the target of one or more trans-acting factor(s) present in the cells undergoing cold acclimation. An additional trans-acting factor is likely targeting the coding region of *cspA* (A_2_A_3_) and is responsible for increasing by almost 3-fold and 1.5- fold the translational activity at 37 °C of D_1_A_2_A_3_ and *cspA* mRNAs, respectively, when the control cell extract is replaced by the cold shock extract (Figure 3d). Unlike at 15 °C, at 37 °C the D_1_A_2_A_3_ mRNA displays a certain degree of translational activity, although it is the least efficient in directing the synthesis of its protein product. This condition might be optimal to emphasize the stimulatory effect of the above-mentioned cold-shock factor(s) targeting the A_2_A_3_ region.

The 30S IC complexes endowed with an overall canonical composition and structure are efficiently and rapidly docked by the 50S ribosomal subunits yielding a functional 70S IC. Because the light scattering analyses showed that 50S docking occurs only with the 30S IC formed with *cspA* mRNA and in part with A_1_A_2_D_3_ mRNA, we can conclude that these are the only mRNAs yielding a canonical 30S IC whereas *cspD*, D_1_A_2_A_3_ and D_1_D_2_A_3_ mRNAs fail to form structurally canonical 30S complexes at low temperature. In this connection, it is not easy to pin down what the structures of the 30S complexes formed with different mRNAs may look like and what can make a given structure look correct or incorrect for the incoming 50S subunit. It is likely that these structures depend upon the structures acquired by the individual mRNAs on the 30S subunit and that some of them may give rise to steric clashes with initiation factor IF3, which overlooks the formation of canonical complexes, or ultimately with the 50S subunit [41]. 

**Figure 6:**
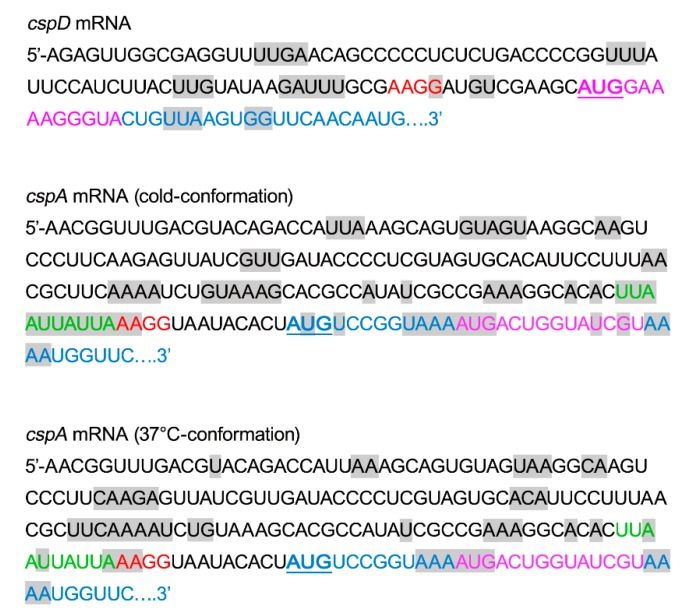
Accessibility of the bases of *cspD* and *cspA* mRNAs to enzymatic and chemical probing. Sequences of the proximal fragments (A_1_A_2_ and D_1_D_2_) of *cspD* and *cspA*, the latter in both its cold- and 37 °C-conformation, as indicated. The initiation triplets AUG are underlined, the SD (red), the AU-rich (green), the coding region (light blue), the DB regions (magenta) and the unpaired bases present in single stranded conformation (highlighted in grey) as deduced from enzymatic and chemical probing on *cspA* mRNA [19] and *cspD* mRNA [29] are indicated.

Previous studies have shown that, unlike *cspA* mRNA which assumes two distinct conformations at 37 °C and at <20 °C [19], the structure of *cspD* mRNA does not change as a function of temperature [29]. This RNA probing showed that the *cspD* mRNA initiation triplet is neither cleaved by enzymes nor modified by chemical reagents which target unpaired bases and is therefore likely buried within a helix. Because both initiation triplet and SD sequence are also sequestered in secondary structures in the 37 °C-conformation of *cspA* mRNA and both *cspA* and *cspD* mRNAs have only a limited number of unpaired nucleotides (Figure 6), these circumstances *per se* do not explain why *cspA* mRNA is well translated and *cspD* mRNA poorly translated at 37 °C. Thus, the main difference between *cspA* and *cspD* mRNAs consists of the presence of an AU-rich sequence immediately upstream the SD sequence of *cspA* mRNA but absent in *cspD* mRNA. It should be noted, in this connection, that this feature of the 5′UTR of *cspA* mRNA is also shared by A_1_D_2_D_3_ and A_1_A_2_D_3_ mRNAs, which are better translated at 37 °C. It is likely that this AU-rich sequence is the binding site for ribosomal protein S1, as suggested by the finding that initiation complex formation on *cspA* mRNA is fully dependent on this protein [19] and that a *cspA* mutant with a deletion in this region of *cspA* mRNA is poorly translated at 37 °C [42]. 

If the presence/absence of the 5′UTR of *cspA* is sufficient to explain the good/poor translational of the mRNAs at 37 °C, this feature alone cannot ensure a good translational activity at low temperature for which also the proximal region of the *cspA* coding sequence is necessary, as shown by our data. In *cspA* mRNA, like in other bacterial mRNAs (e.g., *E. coli lysU*, *glnS*, *rpoH* and several cold-induced *csp* genes) and phage mRNAs (e.g, phage T7 0.3 and 1.0 and phage lambda cI gene), the region immediately downstream of the AUG initiation triplet contains a sequence, known as downstream box (DB), which acts as a translational enhancer [30,31]. In particular, the 14 nts located downstream of the AUG initiation triplet were reported to play a critical role in the expression of cold shock proteins at low temperature [32,33,34,35]. In reality, a DB sequence is also present in *cspD* mRNA [26] immediately downstream the AUG codon in the position indicated in Figure 6. The mechanism initially proposed for the stimulation of translation, namely, a base pairing interaction with an anti-DB sequence (nts 1469–1483) of 16S rRNA [24], proved inconsistent with experimental results demonstrating that such DB base pairing does not occur under normal conditions [43] and after cold stress [44]. In light of these findings which exclude the involvement of the DB sequence in base-pairing with the 16S rRNA, we are left with the hypothesis that the DB in *cspA* and in A_1_A_2_D_3_ mRNAs might be structurally important, by preventing the 5′UTR and the TIR of *cspA* from base pairing with the distal coding region at low temperature. In addition, it is likely the DB region represents the target of CspA itself, a single-stranded RNA binding protein whose level increases dramatically in the cells after cold stress and which might be one of the transacting factors present in the extracts of cold stressed cells which enhance the translational activity of *cspA* and A_1_A_2_D_3_ mRNAs at low temperature. Accordingly, some data demonstrate that CspA specifically stimulates translation at low temperature of its own mRNA folded in its 37 °C-conformation [45]. 

Although it is likely that the structure of 5′UTR and TIR of A_1_A_2_D_3_ mRNA might resemble that of the 37 °C-structure of *cspA* mRNA, it can be excluded that the A_1_A_2_D_3_ mRN*A* could possess a structure similar to that of the cold conformation of *cspA* mRNA which forms 30S IC and is translated much more efficiently than the A_1_A_2_D_3_ mRNA. 

Finally, the 5′UTR of *cspA* has been cloned upstream of the coding region of different target genes with the aim of improving their expression in vivo at low temperature [46]. For some of the target genes, a maximum of a 2-fold improvement was obtained. Our work demonstrates that the 5′UTR alone may not be sufficient to increase the synthesis of a target protein in the cold and that the results will likely depend upon the secondary structures assumed by the resulting chimeric mRNAs. However, the fact that in the presence of cs S30 extracts our chimeric fusion A_1_A_2_D_3_ allowed a high level expression at 15 °C of a toxic protein such as CspD, which cannot be hyper-produced in vivo, seems to be a promising result which paves the way to potential biotechnological applications.

## 4. Materials and Methods

### 4.1. General Preparations

Purified initiation factors (IF1, IF2, and IF3) and 30S and 50S ribosomal subunits were prepared as described previously [47,48]. tRNA_fMet_ was kindly provided by S. V. Kirillov (Gatchina, Russia) and charged and purified as described [49]. mRNAs were prepared by in vitro run-off transcription of the plasmid vectors mentioned below using home-made T7 RNA polymerase essentially as described [13].

### 4.2. Preparation of S30 Fractions

*E. coli* MRE600 cells were grown at 37 °C in LB supplemented with 0.5% glucose. When OD_620_ reached 0.9, one portion of culture was immediately harvested while the remaining half was shifted to 10 °C and was maintained at this cold shock temperature for 120 min. Crude S30 factions were prepared from the cell pellets essentially as described previously [13]. The ribosome content was determined by analytical sucrose density gradient (10%–30%) centrifugation of each crude extract performed in an SW60 rotor using Buffer A (10 mM Tris-HCl, pH 7.7, 60 mM NH_4_Cl, 10 mM Mg acetate, 1 mM DTT). Before use, S30 extracts were incubated for 30 min at 37 °C with 30 mM Tris-HCl, pH 7.7, 10 mM Mg acetate, 75 mM NH_4_Cl, 2 mM DTT, 2 mM ATP, 0.4 mM GTP, 10 mM phosphoenolpyruvate, 0.025 µg of pyruvate kinase/µL reaction, 200 µM of each amino acid, and 0.12 mM citrovorum (Serva) to induce run-off.

### 4.3. Construction of pTZ18A_1_D_2_D_3_, pTZ18D_1_A_2_A_3_, pTZ18A_1_A_2_D_3_ and pTZ18D_1_D_2_A_3_

To swap the 5′-UTR of *cspA* and *cspD* mRNAs, we took advantage of a naturally occurring NlaIII site (5′-CATG↓-3′) which overlaps the initiation codon of *cspD*. In addition, we changed a T to a C immediately upstream of the AUG initiation codon of *cspA* present in pTZ18*cspA* [15] to introduce the NlaIII restriction site also in this position. The site-directed mutagenesis was accomplished using the QuikChange Site-Directed Mutagenesis Kit (Agilent Technologies, Inc., Santa Clara, CA, USA) from Stratagene and the mutagenic primers: 5′-CATTTTACCGGACATGGTGTATTACCTTT-3′ and 5′-AAAGGTAATACACCATGTCCGGTAAAATG-3′. The pTZ18A_1_D_2_D_3_ was obtained by ligating the NlaIII-HindIII-digested pTZ18*cspA* plasmid with the NlaIII-HindIII *cspD* fragment, resulting from the digestion of pTZ18*cspD* [15] and containing the coding region of *cspD*. Similarly, the pTZ18D_1_A_2_A_3_ was obtained by ligating the NlaIII-HindIII-digested pTZ18*cspD* plasmid with the NlaIII-HindIII *cspA* fragment, resulting from the digestion of pTZ18*cspA*. 

To swap the *cspA* and *cspD* sequence immediately downstream of the 9th codon, we performed site-directed mutagenesis so as to introduce a HincII site (5′-GTY↓RAC-3′) on both *cspA* and *cspD* coding regions in pTZ18*cspA* and pTZ18*cspD*. The mutagenic primers were: 5′-GGTACTGTTAAGTGGGTCAACAATGCCAAAGG-3′ and 5′ CCTTTGGCATTGTTGACCCACTTAACAGTACC-3′ for *cspD* and 5′-AATGACTGGTATCGTTAACTGGTTCAACGCTGACAAAGG-3′ and 5-CCTTTGTCAGCGTTGAACCAGTTAACGATACCAGTCATT-3′ for *cspA*. The pTZ18A_1_A_2_D_3_ was obtained by ligating the HincII-HindIII-digested pTZ18*cspA* with the HincII-HindIII *cspD* fragment, resulting from the digestion of pTZ18*cspD*, while the pTZ18D_1_D_2_A_3_ was obtained by ligating the HincII-HindIII-digested pTZ18*cspD* plasmid with the HincII-HindIII *cspA* fragment resulting from the digestion of pTZ18*cspA*.

### 4.4. In Vitro Translation Reactions

In vitro translation reactions were carried out essentially as described [15]. Each reaction mixture (240 µL) contained 160 pmoles of mRNA and the S30 extracts added in an amount corresponding to 80 pmoles of 70S ribosome. The reaction was divided in two parts, one incubated at 37 °C and the other incubated at 15 °C. Samples (15 µL) were withdrawn at the indicated times and spotted on 3MM paper discs for determination of the hot-trichloroacetic acid (TCA)-insoluble radioactivity incorporated.

### 4.5. fMet-tRNA Binding Kinetics

All experiments were performed in Buffer B (20 mM Tris-HCl, pH 7.7, 80 mM NH_4_Cl, 7 mM Mg acetate, 1 mM DTT, 0.5 mM GTP) using a Bio-Logic SFM-400 apparatus (Bio-Logic Science Instruments, Grenoble, France) in quench flow configuration as described [50]. To pre-bind the mRNA to the 30S ribosomal subunit, a mixture (Solution A) containing 1 µM of *E. coli* 30S ribosomal subunits, 1 µM IF1, 1 µM IF2, 1 µM IF3, and 2 µM mRNA, was incubated for 30 min at either 37 °C or 15 °C. To follow the kinetics of fmet-tRNA binding to the pre-formed 30S-IFs-mRNA complex, Syringe A of the quench flow apparatus was filled with Solution A, while syringe B was loaded with 1 uM of f[^35^S]-Met-tRNA. In another set of experiments, to monitor the kinetics of 30S IC formation, Syringe A was filled with a mixture containing 1 µM of *E. coli* 30S ribosomal subunit, 1 µM IF1, 1 µM IF2, and 1 µM IF3 while Syringe B contained 1 µM of f[^35^S] fMet-tRNA and 2 µM of mRNA. The reaction, performed at the indicated temperature, was initiated by rapidly mixing equal volumes (40 µL) of the two solutions, aged for the indicated times and then stopped by rapid dilution and filtration [50]. Manual sampling was used when the binding reaction was incubated for times >15 sec. The amount of f[^35^S]-Met-tRNA bound in the 30S IC was measured by liquid scintillation counting. Apparent rate constants were calculated by fitting the experimental points with (one to three) exponential equations using the Prism Software (Version 5, GraphPad, San Diego, CA, USA). The equations used were: (1) Y=Y_max1_ × (1 − exp(−K_1_ × X)); (2) Y=Y_max1_ × (1 − exp(−K_1_ × X)) + Y_max2_ × (1 − exp(−K_2_ × X)); (3) Y=Y_max1_ × (1 − exp(−K_1_ × X)) + Y_max2_ × (1 − exp(−K_2_ × X)) + Y_max3_ × (1 − exp(−K_3_ × X)).

### 4.6. Light Scattering Measurements

The experiment was carried out in Buffer C (50 mM Tris-HCl pH 7.7, 7 mM Mg acetate, 30 mM KCl, 70 mM NH_4_Cl and 0.5 mM GTP). 30S IC were formed by incubating 0.4 µM of 30S subunits with 0.8 µM of IFs, 0.8 µM of mRNA and 0.6 µM fMet-tRNA at either 37 °C or 15 °C for 30 min. The pre-formed 30S IC (Syringe A) were allowed to mix with 0.4 µM 50S ribosomal subunits (Syringe B) in a Stopped-flow apparatus Kintek SF-2004 (KinTek Corp., Austin, TX). Excitation was at 436 nm and output was monitored with no filtering.

## Figures and Tables

**Table 1:** Apparent rate constants (K_app_) of f[^35^S]-Met-tRNA binding to 30S-IFs-mRNA complexes at the indicated temperatures.

mRNA	37 °C	15 °C
	K_app_ (sec^−1^)	K_app_ (sec^−1^)
*cspA*	106 ± 14	130 ± 29
A_1_A_2_D_3_	160 ± 37	66 ± 10
A_1_D_2_D_3_	120 ± 27	73 ± 24
*cspD*	57 ± 6	51 ± 9
D_1_D_2_A_3_	116 ± 28	34 ± 7
D_1_A_2_A_3_	175 ± 24	53 ± 11

**Table 2:** Apparent rate constants (K_app_) of 30S IC formation monitored by f[^35^S]-Met-tRNA binding at the indicated temperatures.

**37 °C**						
**mRNA**	**K_1app_ (sec^−1^)**	**Y_max1_**	**K_2app_ (sec^−1^)**	**Y_max2_**	**K_3app_ (sec^−1^)**	**Y_max3_**
*cspA*	9.7 ± 3.2	3.0 ± 0.2	0.026 ± 0.003	8.4 ± 3.1	-	-
A_1_A_2_D_3_	9.2 ± 2.9	1.5 ± 0.1	0.017 ± 0.007	2.2 ± 0.8	0.0017 ± 0.0004	7.5 ± 0.7
A_1_D_2_D_3_	11.9 ± 5.7	1.2 ± 0.1	0.016 ± 0.003	4.5 ± 0.8	0.0012 ± 0.0003	8.1 ± 0.5
*cspD*	12.0 ± 5.7	0.96 ± 0.08	0.009 ± 0.002	5.8 ± 1.3	0.00170 ± 0.00065	4.96 ± 1.11
D_1_D_2_A_3_	11.75 ± 3.89	1.64 ± 0.09	0.009 ± 0.002	6.2 ± 1.0	0.0008 ± 0.0005	5 ± 1
D_1_A_2_A_3_	2.50 ± 1.15	0.94 ± 0.01	0.027 ± 0.005	3.8 ± 0.5	0.0012 ± 0.0004	5.2 ± 0.5
**15 °C**						
**mRNA**	**K_1app_ (sec^−1^)**	**Y_max1_**	**K_2app_ (sec^−1^)**	**Y_max2_**	**K_3app_ (sec^−1^)**	**Y_max3_**
*cspA*	-	-	0.07 ± 0.03	3.4 ± 0.8	0.002 ± 0.001	5.3 ± 0.8
A_1_A_2_D_3_	10.0 ± 5.7	0.36 ± 0.05	0.031 ± 0.007	1.35 ± 0.15	0.0006 ± 0.0001	7.2 ± 0.6
A_1_D_2_D_3_	1.1 ± 0.2	0.91 ± 0.05	-	-	0.0003 ± 0.0002	8 ± 5
*cspD*	1.5 ± 0.9	0.24 ± 0.09	0.043 ± 0.003	0.5 ± 0.1	0.0004 ± 0.0003	3.35 ± 1.35
D_1_D_2_A_3_	2.34 ± 1.45	0.16 ± 0.03	0.018 ± 0.004	1.1 ± 0.2	0.0021 ± 0.0005	1.8 ± 0.2
D_1_A_2_A_3_	1.00 ± 0.55	0.54 ± 0.08	-	-	0.0015 ± 0.0005	2.4 ± 0.3
